# An Experimental Investigation on the Flow Boiling Heat Transfer Performance of Nanofluid in 3D Printing Minichannel Heat Sinks: A Comparative Study

**DOI:** 10.3390/nano15141054

**Published:** 2025-07-08

**Authors:** Jianyang Zhou, Zhixin Yin

**Affiliations:** School of Intelligent Manufacturing, Nanning University, Nanning 530004, China

**Keywords:** minichannel heat sink, nanofluid, flow boiling, heat transfer coefficient

## Abstract

A minichannel heat sink combining flow boiling heat transfer with nanofluid is an ideal solution for the long-term cooling of high-power equipment. In the present paper, three mass fractions for 0.01 wt%, 0.05 wt%, and 0.1 wt% graphene/R141b and Al_2_O_3_/R141b nanofluids are prepared by ultrasonic vibration. The flow boiling heat transfer performance for graphene/R141b and Al_2_O_3_/R141b nanofluids was contrastively investigated in a 3D printing 10-minichannel heat sink with a single channel dimension of 198 mm × 1.5 mm × 1.5 mm. The results indicate that the heat transfer performance of graphene/R141b and Al_2_O_3_/R141b nanofluids are enhanced after adding nanoparticles in pure R141b, and the maximum average heat transfer coefficients of graphene/R141b and Al_2_O_3_/R141b nanofluids, respectively, increase by 35.4% and 31.7% compared with that of pure R141b. The heat transfer performance of graphene/R141b and Al_2_O_3_/R141b nanofluids increases nonlinearly with the increase in mass concentration; the heat transfer coefficient reaches its maximum at the mass concentration of 0.02 wt%, and then, it decreases slightly, which is mainly caused by nanoparticle deposition, leading to silted channel surface cavities during the flow boiling experiment. Moreover, it has been discovered that the heat transfer coefficient of graphene/R141b is larger than that of Al_2_O_3_/R141b under the same conditions. The average heat transfer coefficient of graphene/R141b increased by 19.7% compared with that of Al_2_O_3_/R141b. The main reason for this is that graphene nanosheets have a larger contact area with the liquid working medium compared with nanoparticle Al_2_O_3_, and the graphene/R141b thermal conductivity is also significantly higher than that of Al_2_O_3_/R141b nanofluids. The research results can provide a basis for the practical application of nanofluids in heat sinks.

## 1. Introduction

Mini-/microchannel heat sinks have attracted significant research interest due to their excellent heat transfer performance in various cutting-edge fields—semiconductor-based microelectronic components, high-energy laser diode assemblies, and chemical reaction vessels are among some examples [1,2]. However, with the continuous increase in power density, their thermal performance still needs further improvement, making thermal management a major challenge that remains to be addressed.

Many novel surface modification techniques, fabrication technologies, and nanofluid technologies have been applied to heat sinks with miniaturized or micro-scale channels, aiming to enhance heat transfer efficiency even further. The mechanical sanding technique [3], the coating technique [4], direct powder sintering [5], and the 3D printing technique [6] have frequently been utilized for processing these surfaces, and extensive research has been conducted on their heat transfer performances. The primary factors contributing to these improved thermal performances include enhanced bubble dynamics; interactions among the liquid, vapor, and solid phases; and capillary wicking effects. The three-dimensional printing technique has attracted many researchers as a significant alternative to rapid prototyping for heat sinks in the field of heat transfer. One of the main reasons why many researchers use the 3D printing technique as a significant alternative to rapid heat sink prototyping is that 3D printing allows the construction of extremely complex parts in a single manufacturing process, starting from a three-dimensional CAD model, and it does not require customized tooling for each individual part. In the present study, the experimental minichannel heat sink used in this research is fabricated by Direct Metal Laser Sintering, which represents a form of 3D metal printing technology. The main reason for using 3D printing technology is to provide a new technology to enhance flow boiling heat transfer.

Due to the unique characteristics of nanofluids, thermal conductivity can be increased, and heat transfer performance can be optimized through the dispersion of nanoparticles in a base fluid. [7,8,9]. Therefore, the application of nanofluids in the mini-/microchannel heat sinks has been integrated into renewable energy production systems, aiming to improve the thermal performance and increase the energy output of these systems [10,11]. Bahiraei [12,13,14] conducted studies on the flow and heat transfer characteristics of nanofluids and proposed entropy generation prediction models for nanofluid flow in minichannels using neural networks [15]. Boudouh et al. [16] revealed that the flow boiling heat transfer capacity of nanofluids increased with rising nanoparticle concentration in a tiny channel (0.8 mm diameter, 160 mm length). Xu et al. [17] experimentally investigated Al_2_O_3_/H_2_O nanofluid (40 nm diameter, 0.2 wt% concentration) during flow boiling in a rectangular microchannel (0.1 × 0.25 mm, 7.5 mm length), confirming that its heat transfer coefficient far exceeded that of pure water. In recent research, the spotlight has been on carbon-based nanomaterials such as single-walled carbon nanotubes, multi-walled carbon nanotubes, graphene oxide, and graphene nanoplatelets (GNPs) for the fabrication of nanofluids [18,19,20]. Yarmand et al. [20] prepared a GNP-Ag/water nanofluid and analyzed its thermophysical properties and heat transfer performance in circular pipe flow, showing enhanced thermal conductivity and heat transfer rate compared to the base fluid. Wang and Deng [21] used ultrasonic oscillation to fabricate AlN/H_2_O and Al_2_O_3_/H_2_O nanofluids, experimentally studied their saturated flow boiling heat transfer in a vertical tube, and developed a new correlation based on the experimental data. From what has been discussed, it can be concluded that nanofluids can improve the flow boiling heat transfer performance of mini-/microchannel heat sinks. However, limited research in the comparative investigation of graphene/R141b and Al_2_O_3_/R141b nanofluid flow boiling heat transfer performance in 3D printing mini-/microchannels heat sinks has been reported.

In the present study, three mass fractions for 0.01 wt%, 0.05 wt%, and 0.1 wt% graphene/R141b and Al_2_O_3_/R141b nanofluids are prepared by ultrasonic vibration. Graphene nanofluid is chosen because of its advantages in heat transfer, and graphene/R141b nanofluids have never been investigated in 3D printing mini-/microchannels heat sinks. The main objective of this investigation is to compare the flow boiling heat transfer performance of two kinds of nanofluids, and the research can provide a basis for the practical application of nanofluids in heat sinks.

## 2. Experiments

### 2.1. Experimental Apparatus and Procedure

**Figure 1:**
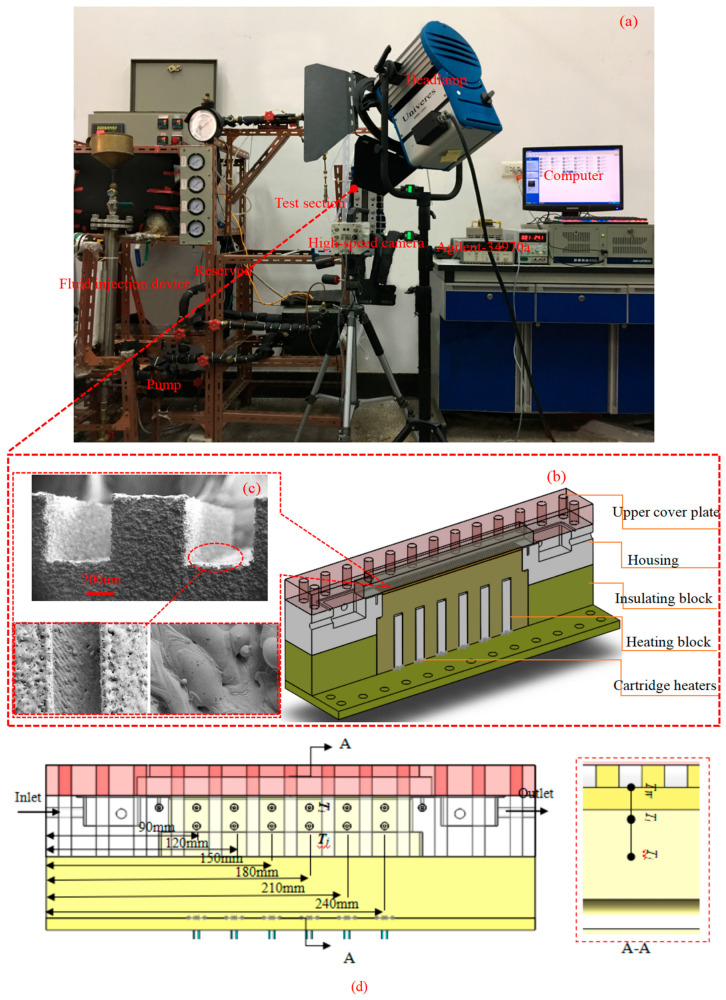
(**a**–**d**) Schematic diagram of the experimental apparatus.

The experimental setup is illustrated schematically in Figure 1a. The experimental configuration employed in this study mirrored those documented in References [22,23,24]. The liquid working medium was stored in a reservoir. The liquid working medium was pumped through a filter. A flow meter was used to measure the flow of working medium passing through the heat sink. The minichannel heat sink was heated using six cartridge heaters with a power rating of 900 W. The output current and voltage of the electric hot wire were regulated by a voltage regulator, and a multimeter was used to measure the output power. Two thermocouples recorded the inlet and outlet temperatures of the test section. A pressure difference transducer was installed between two nozzles to measure the pressure drop. All generated signals, including thermocouple temperatures, pressure signals, and flow rate signals, were recorded by an Agilent-34970A data acquisition instrument. The internal pressure of the system could be adjusted via the cooling system. All sections were wrapped with insulating cotton to minimize heat losses. The heat sink consisted of ten minichannels with dimensions of 1.5 mm in width, 1.5 mm in height, and 195 mm in length. The test section included a visual glass plate, heating block, flow housing, cartridge heaters, and cover plate (see Figure 1b for details). The distance was 8.61 mm between the minichannel bottom wall (*T*_W_) and the upper wall temperature (*T_i_*). In conclusion, the data collection model is shown in Figure 1d. Figure 1a represents the experimental system, and Figure 1b,c represent the three-dimensional structure of the test experimental section and the surface structure SEM of the minichannel heat sink.

In the present work, the minichannel heat sink is fabricated by Direct Metal Laser Sintering, which has been illustrated in Refs. [22,24]. DMLS belongs to a branch of the 3D printing technique. The geometric dimensions of the heat sink are divided into layered units in the computer. The part’s cross-sections (layers) are sequentially filled with elongated molten powder lines (vectors). Each sintering layer has a thickness of 0.03 mm. The specimen material property is stainless steel. After the implementation of laser sintering for fabrication, the heat sink samples are carefully cleansed. Then, the surface features of the fabricated heat sinks are characterized via Scanning Electron Microscopy (SEM), as demonstrated in Figure 1c.

After the experimental apparatus is built, the experimental system’s accuracy must be examined. The detailed process is as follows: firstly, *Q_l_* is defined as the heat absorbed by the working fluid. It should be ensured that there is no phase change for the liquid working medium R141b. Additionally, the input power produced from the cartridge heaters is defined as *Q*. The heat loss deviation has the following definition:

(1) $$\varepsilon =\frac{\left|Q-Q_{l}\right|}{Q}\times 100\%$$

(2) $$Q_{l}=Mc_{f}\left(T_{\mathrm{out}}-T_{\mathrm{in}}\right)$$

It was shown by the results that the heat loss deviation fluctuated in the range of 8%–15% under diverse mass flux conditions. As soon as the experimental heat flux exceeded 7 kW∙m^−2^, the heat loss deviation remained roughly stable, signifying that the experimental system met the accuracy specifications. The feasibility of this method has been verified by the references [25,26].

In the saturated region, the heat transfer coefficient is determined through an energy balance analysis in the single channel [1]. Here, *T*_sat_ represents the saturated temperature, while *T*_w_ stands for the temperature of the bottom channel wall.

(3) $$q\left(W_{\mathrm{ch}}+W_{w}\right)=h(T_{w}-T_{\mathrm{sat}})\left(W_{\mathrm{ch}}+2\eta H_{\mathrm{ch}}\right)$$

(4) $$T_{w}=T_{i}-qR_{t}$$

The heat flux can be expressed as

(5) $$q=-k_{al}\frac{T_{j}-T_{i}}{7.0}$$

(6) $$R_{t}=(\frac{7.0}{k_{\mathrm{al}}}+\frac{1.5}{k_{\mathrm{st}}}+\frac{0.11}{k_{\mathrm{dr}}})\times 10^{-3}$$

where *k*_al_ is the thermal conductivity of the aluminum base, *k*_st_ is the thermal conductivity of the heat sink, and *k*_dr_ is the thermal conductivity of thermal silica. *A* is the heating area for the minichannel. *η* is the fin efficiency for a rectangular channel. *W*_w_ is the distance between the two fins. H_ch_ is the height of the fin.

(7) $$\eta =\frac{\tanh(mH_{\mathrm{ch}})}{mH_{\mathrm{ch}}}$$

(8) $$m=\sqrt{\frac{2h}{k_{\mathrm{al}}W_{w}}}$$

Evaluating the maximum deviation of measured values is crucial, and it should be based on the apparatus accuracy. A deviation analysis was performed according to the method proposed by Moffat [27]. The maximum deviation of a dependent variable *k_xi_*_,max_ is defined as in Equation (9).

(9) $$\varphi_{\max,x_{i}}=\frac{\Delta \varphi }{\varphi_{\min}}\times 100\%$$

wherein ∆*φ* is the unit scale value of the measuring instrument and *k*_min_ is the minimum of the measured physical variables. For *y*(*xi*) with independent linear parameters (e.g., *y(xi) = y*(*x*1, *x*2, *∙∙∙, xi*)), the error transfer principle shows that the maximum deviation of *y(xi)* can be derived from Equation (10). In this paper, the maximum deviations of *q, T_w_*, and *h* are 1.0%, 1.02%, and 1.42%, respectively.

(10) φmax,y(xi)=φmax,x12+φmax,x22+⋅⋅⋅+φmax,xn212×100%

### 2.2. Preparation and Stability of Nanofluids

**Figure 2:**
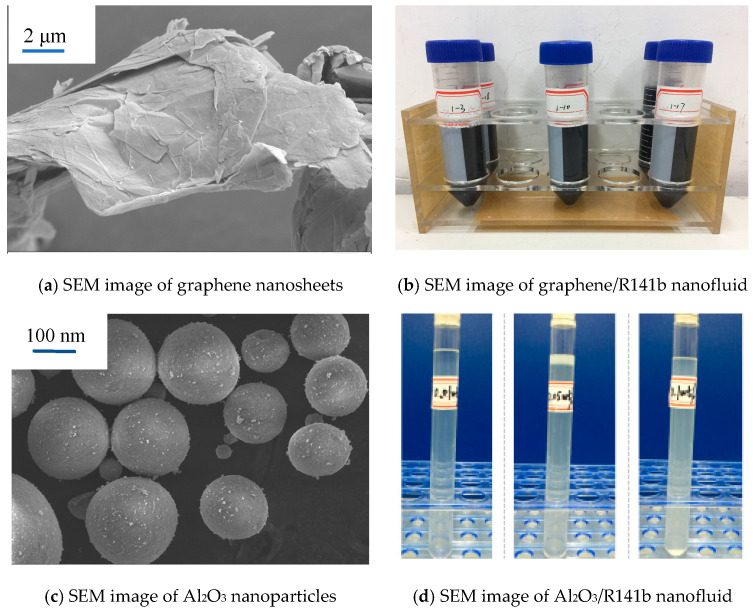
Two different types of nanofluids.

Graphene/R141b and Al_2_O_3_/R141b nanofluids are prepared by ultrasonic vibration. The graphene nanosheet and Al_2_O_3_ nanoparticles used in the experiment were purchased. SEM pictures of graphene and Al_2_O_3_ nanoparticles are shown in Figure 2. Firstly, a certain portion of nanoparticles were diluted with refrigerant R141b into a nanofluid with the required concentration by ultrasonic vibration for one hour. Figure 2b,d show different mass fractions (0.01%, 0.05%, and 0.1%) of graphene/R141b and Al_2_O_3_/R141b nanofluids. In Figure 2b, numbers 1–3, 1–10, and 1–17, respectively, represent the mass fractions of 0.01%, 0.05%, and 0.1% graphene/R141b nanofluids. Table 1 indicates the graphene/R141b and Al_2_O_3_/R141b nanofluid physical parameters at a saturated temperature.

After graphene/R141b and Al_2_O_3_/R141b nanofluids had been prepared, graphene/R141b and Al_2_O_3_/R141b nanofluid zeta potentials were characterized using a HORIBA Nanoparticle Analyzer SZ-100. The results show that the zeta potential average value of different mass fractions (0.01%, 0.05%, and 0.1%) of graphene/R141b and Al_2_O_3_/R141b nanofluids were in the range of 35.3 mV to 42.2 mV, and the zeta potential average value were in the range of 32.1 mV to 41.8 mV in a second test (after 20 days). According to colloidal theory [28], when the absolute value of zeta potential exceeds 30 mV, nanofluids can maintain sufficient repulsive forces between particles. Meanwhile, the stability of prepared graphene/R141b and Al_2_O_3_/R141b nanofluids was confirmed by the absence of sedimentation after being kept stationary for 20 days. Therefore, the good dispersion stability of different mass fractions (0.01%, 0.05%, and 0.1%) of graphene/R141b and Al_2_O_3_/R141b nanofluids is obtained in this study.

The physical parameters of nanofluids can be calculated according to Equations (11)–(15) [29]. Sommers and Yerkes [30] experimentally discovered that the maximum deviation between the referenced correlation of physical properties and experimental date is less than 3%, which indicates the physical properties of different mass fractions (0.01%, 0.05%, 0.1%) of graphene/R141b and Al_2_O_3_/R141b nanofluids; they are available in this article.

(11) $$\varphi_{\mathrm{np},l}=\frac{w\rho_{l}}{(1-w)\rho_{np}+w\rho_{l}}$$

(12) $$\rho_{\mathrm{np},l}=(1-\varphi )\rho_{l}+\varphi \rho_{\mathrm{np}}$$

(13) $$\lambda_{\mathrm{np},l}=\frac{\lambda_{\mathrm{np}}+2\lambda_{l}-2(\lambda_{l}-\lambda_{\mathrm{np}})\varphi }{\lambda_{\mathrm{np}}+2\lambda_{l}+(\lambda_{l}-\lambda_{\mathrm{np}})\varphi }\lambda_{l}$$

(14) $$\mu_{\mathrm{np},l}=\mu_{l}(1+2.5\varphi +6.25\varphi^{2})$$

(15) $$c_{\mathrm{np},l}=(1-\varphi )c_{l}+\varphi c_{\mathrm{np}}$$

where *φ*, *w*, *ρ*, *c*, and *μ* are volume fraction, mass fraction, density, specific heat capacity, and viscosity, respectively. The subscripts *np.l*, *l*, and *np* stand for nanofluid, R141b, and nanoparticle, respectively.

## 3. Experimental Results and Discussions

**Figure 3:**
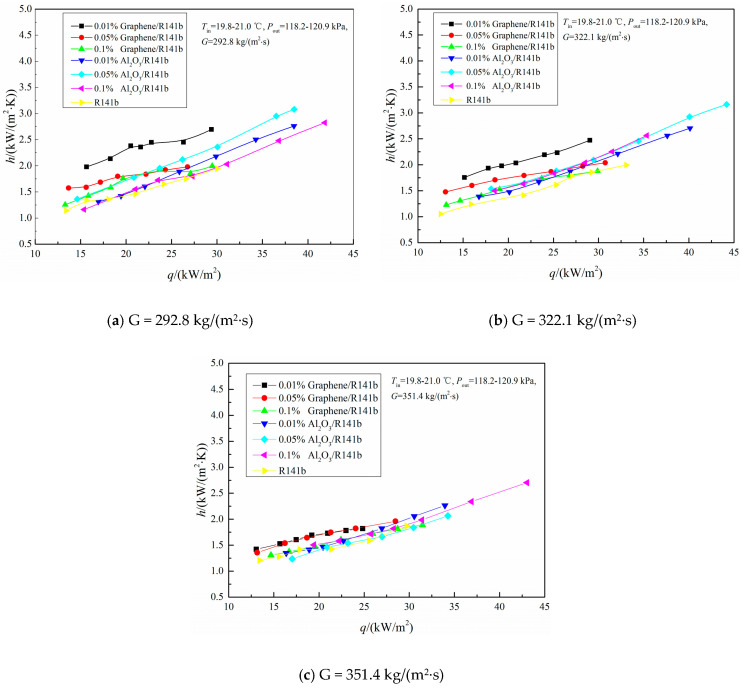
Variation in heat transfer coefficient with heat flux.

As depicted in Figure 3, the changes in the local heat transfer coefficient of the 0.025 wt%, 0.05 wt%, and 0.1 wt% graphene/R141b and Al_2_O_3_/R141b nanofluids are illustrated as the heat flux increases. These observations were made under mass flow rates of 292.8 kg/(m^2^∙s), 322.1 kg/(m^2^∙s), and 351.4 kg/(m^2^∙s) within 3D printing heat sinks. The local heat transfer coefficients of 0.01 wt%, 0.05 wt%, and 0.1 wt% graphene/R141b and Al_2_O_3_/R141b nanofluids increase in direct proportion to the heat flux. Compared to the pure refrigerant R141b, the heat transfer performance of both graphene/R141b and Al_2_O_3_/R141b nanofluids is enhanced under the mass flow rates of 292.8 kg/(m^2^∙s), 322.1 kg/(m^2^∙s), and 351.4 kg/(m^2^∙s). For instance, when the mass flow rate G = 292.8 kg/(m^2^∙s) (refer to Figure 3a for more details), the average heat transfer coefficients of 0.01 wt%, 0.05 wt%, and 0.1 wt% graphene/R141b nanofluids increase by 35.4%, 14.0%, and 10.6%, respectively, relative to that of the pure refrigerant R141b. Similarly, the average heat transfer coefficients of 0.01 wt%, 0.05 wt%, and 0.1 wt% Al_2_O_3_/R141b nanofluids increase by 22.1%, 31.7%, and 21.5%, respectively, compared to the pure refrigerant R141b. The experimental findings clearly indicate that incorporating nanoparticles (graphene nanosheets and Al_2_O_3_ nanoparticles) into the pure refrigerant R141b can improve its heat transfer performance.

**Figure 4:**
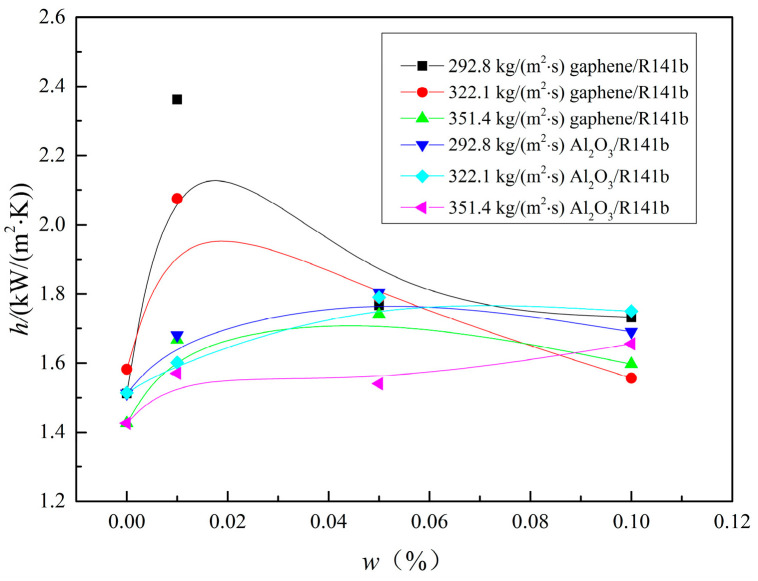
The trend of the heat transfer coefficient as a function of mass concentration.

As can be seen from Figure 4, the variation in the local heat transfer coefficient (*h5*) of the graphene/R141b and Al_2_O_3_/R141b nanofluids with mass concentrations indicates that, under the same circumstances, the heat transfer performance exhibits a nonlinear increase as the mass concentration goes up. The peak of the heat transfer coefficient is achieved at a mass concentration of 0.02 wt%, and then it slightly decreases. References [31,32] have obtained comparable research results showing that the heat transfer coefficients increase with the increase in mass concentrations in the low concentration range. References [24,33] have also found that there is an optimal nanoparticle concentration for the best heat transfer enhancement, because the deposition of nanoparticles will lead to a deterioration of the heat transfer performance. At the same time, the heat transfer performance of graphene/R141b nanofluids is better than that of Al_2_O_3_/R141b nanofluids. Through a comparative study, it is found that the flow boiling heat transfer coefficient of graphene/R141b refrigerant nanofluids is 8.0–39.8% higher than that of Al_2_O_3_/R141b nanofluids.

From Figure 3 and Figure 4, the experiment results show that the heat transfer performance in the 3D printing minichannel heat sink is enhanced after adding graphene and Al_2_O_3_ in pure refrigerant R141b. The maximum average heat transfer coefficients of graphene/R141b nanofluids increase by 35.4% compared with the pure refrigerant R141b. The maximum average heat transfer coefficients of Al_2_O_3_/R141b nanofluids, respectively, increase by 31.7% compared with the pure refrigerant R141b. The main reasons are listed below. Firstly, the suspended nanoparticles enhance the effective thermal conductivity of the fluid, resulting in a higher thermal conductivity compared to the base fluid (refer to Table 1 for specific information). Secondly, the Brownian motion of the nanoparticles boosts the mixing fluctuations and turbulence within the fluid. For a fully developed, steady-state nanofluid, research has indicated that the particle mass balance conforms to Equation (16) [34].

(16) $$J+r\frac{dJ}{dr}=0$$

Here, *r* represents the radial coordinate, and *J* is the total particle flux in the *r* direction. As previously stated, the total flux of particle migration is composed of three components: the particle fluxes generated by the viscosity gradient *J_μ_*, the non-uniform shear rate *J_c_*, and Brownian motion *J_b_*. The particle fluxes derived from the aforementioned three terms induce the migration of particles within the fluid, particularly during flow boiling heat transfer. This migratory movement promotes better heat exchange between the nanoparticles and the fluid. As a result, the heat transfer performance of the nanofluid is augmented.

(17) $$J_{\mu }=-k_{\mu }\overset{\cdot }{\gamma }\varphi^{2}(\frac{D_{\mathrm{np}}^{2}}{\mu_{l}})\frac{d\mu_{l}}{d\varphi }\nabla \varphi$$

(18) $$J_{c}=-k_{c}D_{\mathrm{np}}^{2}(\varphi^{2}\nabla \dot{\gamma }+\varphi \dot{\gamma }\nabla \varphi )$$

(19) $$J_{b}=-\frac{k_{B}T}{3\pi \mu_{l}D_{\mathrm{np}}}\nabla \varphi$$

where *k_μ_* and *k_c_* are constants, *k_B_* is the Boltzmann constant, *φ* represents volume fraction concentration, $\overset{\cdot }{\gamma }$ is the shear rate, *T* denotes the temperature, *D*_np_ indicates the nanoparticle diameter, and μL is the viscosity. Reference [32] also reported analogous findings. Nanoparticles can improve heat transfer performance because the interactions between particles enhance the turbulence intensity of the fluid. Another key factor contributing to the improved heat transfer performance is the thinning of the liquid film, which reduces the movement space for nanoparticles within the film. Nanoparticles adhere to the channel bottom, forming a nanoparticle layer—both phenomena collectively enhance the thermal conductivity of the heat transfer surface as described. Additionally, the heating surface continuously transfers heat, causing the liquid film in the micro-liquid layer to evaporate steadily. This evaporation drives the triple-phase line to move leftward, decreasing surface tension at the heat transfer surface and increasing bubble departure frequency. Collectively, these mechanisms enhance the overall heat transfer performance.

However, when the mass concentration exceeds 0.02 wt%, the heat transfer coefficients of the graphene/R141b and Al_2_O_3_/R141b nanofluids decline with increasing concentration, primarily due to nanoparticle deposition on the channel surface during flow boiling experiments. Ahmed et al. [33] experimentally demonstrated that nanoparticle deposition significantly affects heat transfer: particle accumulation clogs cavities, filling them with nanoparticles. According to Hsu’s theory [35], these cavities cannot act as bubble embryos because vapor is not trapped at the cavity base when liquid flows through. After nanofluid flow boiling, nanoparticles fill the cavities on 3D-printed minichannel surfaces, preventing them from serving as nucleation sites and reducing the activated cavity density per unit area. This weakens heat transfer performance, causing heat transfer coefficients to decrease as mass concentration increases from 0.02 to 0.1 wt% at G = 292.8 and 322.1 kg/(m^2^·s) and from 0.035 to 0.1 wt% at G = 351.4 kg/(m^2^·s).

In addition, it is interesting to observe that the local heat transfer coefficient of graphene/R141b is larger than that of Al_2_O_3_/R141b under the same conditions. Taking G = 292.8 kg/(m^2^∙s) conditions as an example, system pressure ranges from 118.2 kPa to 120.9 kPa, and inlet temperature ranges from 19.8 °C to 21.0 °C, respectively. The heat transfer coefficient of 0.01 wt%, 0.05 wt%, and 0.1 wt% graphene/R141b increased by 39.8%, 8.0%, and 11.3% compared with that of 0.01wt%, 0.05 wt%, and 0.1 wt% Al_2_O_3_/R141b, respectively. The average heat transfer coefficient of graphene/R141b increased by 19.7% compared with that of Al_2_O_3_/R141b. The main reason is that graphene nanosheets have a larger contact area with the liquid working medium compared with nanoparticle Al_2_O_3_. Therefore, there is a better heat transfer performance for graphene nanofluid compared with that of Al_2_O_3_/R141b nanofluids. In addition, the thermal conductivity of graphene nanosheets is superior to that of Al_2_O_3_ nanoparticles. From Table 1, graphene/R141b thermal conductivity is also significantly higher than that of Al_2_O_3_/R141b nanofluids, and graphene/R141b thermal conductivity is 6.43 times that of Al_2_O_3_/R141b nanofluid. Therefore, more heat was taken away in the flow boiling process.

## 4. Conclusions

In the present study, three mass fractions for 0.01 wt%, 0.05 wt%, and 0.1 wt% graphene/R141b and Al_2_O_3_/R141b nanofluids can be synthesized via ultrasonic vibration. Two different nanofluids’ flow boiling heat transfer performances were studied by an experimental comparative investigation of 3D printing minichannels heat sinks. Key findings are outlined below.

(1) Experimental results indicate that introducing nanoparticles into pure R141b fluid enhances its heat transfer performance. Compared to pure R141b, the maximum average heat transfer coefficients of graphene/R141b and Al_2_O_3_/R141b nanofluids increase by 35.4% and 31.7%, respectively. The heat transfer performance of these nanofluids exhibits nonlinear growth with increasing mass concentration: it peaks at 0.02 wt% and then slightly declines.
(2) It is interesting to observe that the heat transfer coefficient of graphene/R141b is larger than that of Al_2_O_3_/R141b under the same conditions. The average heat transfer coefficient of graphene/R141b increased by 19.7% compared with that of Al_2_O_3_/R141b. The main reason is that graphene nanosheets have a larger contact area with the liquid working medium compared with the nanoparticle Al_2_O_3_, and the graphene/R141b thermal conductivity is also significantly higher than that of Al_2_O_3_/R141b nanofluids.
(3) The key factor behind the improved heat transfer performance is that suspended nanoparticles enhance the fluid’s effective thermal conductivity, making it higher than that of the base fluid. Furthermore, the Brownian motion of nanoparticles intensifies mixing fluctuations and turbulence within the fluid. Additionally, the liquid film thickness thins, reducing the movement space of nanoparticles in the film. For graphene/R141b and Al_2_O_3_/R141b nanofluids, their heat transfer coefficients decrease as mass concentration increases, primarily due to nanoparticle deposition on the channel surface during flow boiling experiments.

## Figures and Tables

**Table 1:** Physical parameters of nanofluids.

	*w*/%	*ρ*_np,_*_l_*/(kg·m^−3^)	*c*_np,_*_l_*/(kJ·kg^−1^·K^−1^)	*λ*_np,_*_l_*/(W·m^−1^·K^−1^)	*μ*_np,_*_l_* × 10^−3^/(Pa·s)
Al_2_O_3_/R141b	0.01%	1186.708	1.188	0.454	0.315
	0.05%	1186.716	1.186	0.454	0.315
	0.1%	1186.733	1.186	0.453	0.316
Graphene/R141b	0.01%	1186.703	1.188	2.908	0.315
	0.05%	1186.716	1.189	2.910	0.315
	0.1%	1186.733	1.189	2.912	0.316

## Data Availability

Data are contained within the article.

## Nomenclature

*A*	Minichannel heating area
*c_f_*	Liquid specific heat, J/(kg∙K)
*M*	Mass flow rate, kg/s
*G*	Mass flux, kg/(m^2^∙s)
*h*_fg_	Latent heat of vaporization, J/kg
*H*_ch_	Channel depth, m
*k*_al_	Aluminum thermal conductivity, W/(m∙K)
*k*_st_	Heat sink thermal conductivity, W/(m∙K)
*k_dr_*	Silica thermal conductivity, W/(m∙K)
*q*	Heat flux, kW/m^2^
*Q*	Input power produced, W
*Q*_l_	Heat absorbed by working fluid, W
*R*_t_	Thermal conductivity of copper, W/(m∙K)
*T*_w_	Wall temperature, °C
*T*_sat_	Fluid saturation temperature, °C
*T_j_*	Upper wall temperature, °C
*T_i_*	Bottom wall temperature, °C
*W*_w_	Minichannel interval spacing, m
*W*_ch_	Channel spacing, m
*Greeks*	
*μ*_np,_ *_l_*	Liquid viscosity, Pa.s
*ρ*_np,_ *_l_*	Liquid density, kg/m^3^
*c*_np,_ *_l_*	Specific heat capacity, kJ∙kg^−1^∙K^−1^
*λ*_np,_ *_l_*	Thermal conductivity, W∙m^−1^∙K^−1^

## References

[1] Lee H., Park I., Mudawar I., Hasan M.M. (2014). Micro-channel evaporator for space applications-1. Experimental pressure drop and heat transfer results for different orientations in earth gravity. Int. J. Heat Mass Transf..

[2] Mudawar I., Bharathan D., Kelly K., Narumanchi S. (2009). Two-phase Spray Cooling of Hybrid Vehicle Electronics. IEEE Trans. Compon. Packag. Technol..

[3] Sadaghiani A.K., Saadi N.S., Parapari S.S., Karabacak T., Keskinoz M., Koşar A. (2017). Boiling heat transfer performance enhancement using micro and nano structured surfaces for high heat flux electronics cooling systems. Appl. Therm. Eng..

[4] Li S.-F., Bao Y.-Y., Wang P.-Y., Liu Z.-H. (2018). Effect of nano-structure coating on thermal performance of thermosyphon boiling in micro-channels. Int. J. Heat Mass Transf..

[5] Wei W., Deng D., Huang Q., Zeng T., Huang Y. (2017). Experimental study and optimization of pin fin shapes in flow boiling of micro pin fin heat sinks. Appl. Therm. Eng..

[6] Wonga M., Owen I., Sutcliffe C.J., Puri A. (2009). Convective heat transfer and pressure losses across novel heat sinks fabricated by Selective Laser Melting. Int. J. Heat Mass Transf..

[7] Saidur R., Kazi S., Hossain M., Rahman M., Mohammed H. (2011). A review on the performance of nanoparticles suspended with refrigerants and lubricating oils in refrigeration systems. Renew. Sustain. Energy Rev..

[8] Patil M., Kim S., Seo J.H., Lee M.Y. (2015). Review of the Thermo-Physical Properties and Performance Characteristics of a Refrigeration System Using Refrigerant-Based Nanofluids. Energies.

[9] Peng H., Lin L., Ding G. (2015). Influences of primary particle parameters and surfactant on aggregation behavior of nanoparticles in nanorefrigerant. Energy.

[10] Abdollahi A., Mohammed H.A., Vanaki S.M., Sharma R.N. (2018). Numerical investigation of fluid flow and heat transfer of nanofluids in microchannel with longitudinal fins. Ain Shams Eng. J..

[11] Wu J., Zhao J., Lei J., Liu B. (2016). Effectiveness of nanofluid on improving the performance of microchannel heat sink. Appl. Therm. Eng..

[12] Bahiraei M. (2016). Particle migration in nanofluids: A critical review. Int. J. Therm. Sci..

[13] Bahiraei M., Hangi M. (2015). Flow and heat transfer characteristics of magnetic nanofluids: A review. J. Magn. Magn. Mater..

[14] Bahiraei M., Abdi F. (2016). Development of a model for entropy generation of water-TiO_2_ nanofluid flow considering nanoparticle migration within a minichannel. Chemom. Intell. Lab. Syst..

[15] Bahiraei M., Majd S.M. (2016). Prediction of entropy generation for nanofluid flow through a triangular minichannel using neural network. Adv. Powder Technol..

[16] Boudouh M., Gualous H.L., Labachelerie M.D. (2010). Local convective boiling heat transfer and pressure drop of nanofluid in narrow rectangular channels. Appl. Therm. Eng..

[17] Xu L., Xu J. (2012). Nanofluid stabilizes and enhances convective boiling heat transfer in a single microchannel. Int. J. Heat Mass Transf..

[18] Safaei M., Ahmadi G., Goodarzi M., Safdari Shadloo M., Goshayeshi H., Dahari M. (2016). Heat Transfer and Pressure Drop in Fully Developed Turbulent Flows of Graphene Nanoplatelets-Silver/Water Nanofluids. Fluids.

[19] Yarmand H., Gharehkhani S., Ahmadi G., Shirazi S.F.S., Baradaran S., Montazer E., Zubir M.N.M., Alehashem M.S., Kazi S., Dahari M. (2015). Graphene nanoplatelets–silver hybrid nanofluids for enhanced heat transfer. Energy Convers. Manag..

[20] Yarmand H., Gharehkhani S., Shirazi S.F.S., Goodarzi M., Amiri A., Sarsam W.S., Alehashem M.S., Dahari M., Kazi S.N. (2016). Study of synthesis, stability and thermo-physical properties of graphene nanoplatelet/platinum hybrid nanofluid. Int. Commun. Heat Mass Transf..

[21] Wang Y., Deng K.H., Liu B., Wu J.M., Su G.H. (2017). A correlation of nanofluid flow boiling heat transfer based on the experimental results of AlN/H_2_O and Al_2_O_3_/H_2_O nanofluid. Exp. Therm. Fluid Sci..

[22] Zhou J., Luo X., Pan Y., Wang D., Xiao J., Zhang J., He B. (2019). Flow boiling heat transfer coefficient and pressure drop in minichannels with artificial activation cavities by direct metal laser sintering. Appl. Therm. Eng..

[23] Zhou J., Luo X., Feng Z., Xiao J., Zhang J., Guo F., Li H. (2017). Saturated flow boiling heat transfer investigation for nanofluid in minichannel. Exp. Therm. Fluid Sci..

[24] Zhou J., Luo X., Deng C., Xie M., Zhang L., Wu D., Guo F. (2017). Influence of nanoparticle concentrations on flow boiling heat transfer coefficients of Al_2_O_3_/R141b in micro heat exchanger by direct metal laser sintering. Chin. J. Chem. Eng..

[25] Yong T., Chen C., Zhang S., Sun Y., Zeng J., Yuan W., Li Z. (2016). Effects of structural parameter on flow boiling performance of interconnected microchannel net. Appl. Therm. Eng..

[26] Deng D., Xie Y., Huang Q., Wan W. (2017). On the flow boiling enhancement in interconnected reentrant microchannels. Int. J. Heat Mass Transfer..

[27] Moffat R.J. (1988). Describing the uncertainties in experimental results. Exp. Therm. Fluid Sci..

[28] Si Y., Samulski E.T. (2008). Synthesis of water soluble graphene. Nano Lett..

[29] Kandlikar S.G., Grande W.J. (2003). Evolution of Microchannel Flow Passages--Thermohydraulic Performance and Fabrication Technology. Heat Transf. Eng..

[30] Sommers A.D., Yerkes K.L. (2010). Experimental investigation into the convective heat transferand system-level effects of Al_2_O_3_-propanol nanofluid. J. Nanoparticle Res..

[31] Yang X.-F., Liu Z.-H. (2012). Flow boiling heat transfer in the evaporator of a loop thermosyphon operating with CuO based aqueous nanofluid. Int. J. Heat Mass Transf..

[32] Prajapati O.S., Rohatgi N. (2014). Flow Boiling Heat Transfer Enhancement by Using ZnO-Water Nanofluids. Sci. Technol. Nucl. Install..

[33] Ahmed O., Hamed M. (2012). Experimental investigation of the effect of particle deposition on pool boiling of nanofluids. Int. J. Heat Mass Transf..

[34] Ding Y., Wen D. (2005). Particle migration in a flow of nanoparticle suspensions. Powder Technol..

[35] Hsu Y.Y. (1962). On the Size Range of Active Nucleation Cavities on a Heating Surface. J. Heat Transf..

